# Body Weight Can Change How Your Emotions Are Perceived

**DOI:** 10.1371/journal.pone.0166753

**Published:** 2016-11-21

**Authors:** Yujung Oh, Norah C. Hass, Seung-Lark Lim

**Affiliations:** Department of Psychology, University of Missouri – Kansas City, Kansas City, Missouri, United States of America; Radboud Universiteit, NETHERLANDS

## Abstract

Accurately interpreting other’s emotions through facial expressions has important adaptive values for social interactions. However, due to the stereotypical social perception of overweight individuals as carefree, humorous, and light-hearted, the body weight of those with whom we interact may have a systematic influence on our emotion judgment even though it has no relevance to the expressed emotion itself. In this experimental study, we examined the role of body weight in faces on the affective perception of facial expressions. We hypothesized that the weight perceived in a face would bias the assessment of an emotional expression, with overweight faces generally more likely to be perceived as having more positive and less negative expressions than healthy weight faces. Using two-alternative forced-choice perceptual decision tasks, participants were asked to sort the emotional expressions of overweight and healthy weight facial stimuli that had been gradually morphed across six emotional intensity levels into one of two categories—“neutral vs. happy” (Experiment 1) and “neutral vs. sad” (Experiment 2). As predicted, our results demonstrated that overweight faces were more likely to be categorized as happy (i.e., lower happy decision threshold) and less likely to be categorized as sad (i.e., higher sad decision threshold) compared to healthy weight faces that had the same levels of emotional intensity. The neutral-sad decision threshold shift was negatively correlated with participant’s own fear of becoming fat, that is, those without a fear of becoming fat more strongly perceived overweight faces as sad relative to those with a higher fear. These findings demonstrate that the weight of the face systematically influences how its emotional expression is interpreted, suggesting that being overweight may make emotional expressions appear more happy and less sad than they really are.

## Introduction

Body weight has become a global focal point as obesity rates increase in many countries. Within the United States alone, over one-third of the adult population has been categorized as obese [1], making this attention on weight and the consequences thereof both unsurprising and strongly warranted. According to the evidence report by the National Heart, Lung and Blood Institute [2], the health costs of being overweight can be quite severe, including an increased risk of Type 2 diabetes, stroke, and heart disease. In addition to having serious implications on physical well-being, the social consequences of being overweight also have a significant, although often overlooked, impact on one’s life. Research has shown that overweight individuals experience many forms of social discrimination [3]. There is evidence that overweight job applicants are less likely to be hired than healthy weight individuals and that they are paid less and perceived as less qualified, less likely to succeed, and having poorer leadership skills [4–7]. It has also been found that being overweight can lead one to be rated as less attractive, less trustworthy, or less competent [8–10]. The consequences of being overweight have been shown to permeate through different social contexts, with negative weight-stigma experiences found in interactions between friends, spouses, and even parents [11]. Psychologically, being overweight or obese, a term reserved for those significantly overweight, is highly linked to negative mental health outcomes such as depression, poor self-esteem and increased perceived stress [12–14]. Thus, it is apparent that the consequences of being overweight can have a significant negative impact on one’s social, physical, and psychological functioning.

Although being overweight is often correlated with negative psychosocial consequences, a predominant stereotyped perception somewhat ironically associates being overweight with happiness, contentment, laziness, or carefree attitudes. In William Shakespeare’s classic tragedy of Julius Caesar, Act 1, a fat man is described as a contented person, while a lean man is described as a hungry, dangerous person. Similarly, in current American television shows, heavier-weight characters are often portrayed as the objects of ridicule [15]. Crisp and colleagues [16, 17] even coined the term “jolly fat” to capture the idea of the association between being overweight and being happy when they found that an overweight, middle-aged suburban and rural population reported low levels of anxiety and depression. There was further support for the “jolly fat” hypothesis in other population-based studies, which found that higher body mass index (BMI) was inversely associated with depression scores in an older population [18, 19]. However, it has been pointed out that overweight adults might be less likely to reveal negative emotions to others due to social desirability bias [16] or cultural values [19], or that appetite might play a moderating role on the relationship between obesity and depression [18].

As mentioned above, though, being overweight has more recently been linked to increased depression [20], making stereotypes implying the opposite counterintuitive. Although the exact origin of the social stereotype of overweight individuals as jolly and happy is unknown, it may be due in part from the socially prevailing idea that a carefree, indulgent lifestyle leads to bliss, but might also lead to overindulgence and obesity. A study that investigated adolescent’s attitudes towards their health lifestyle with a focus on overweightness reported that participants held a general lack of interest in their future health but an overall attitude of living to enjoy the moment [21]. Other research measuring implicit weight bias found higher implicit anti-fat attitudes when overweight individuals were portrayed as participating in stereotype consistent activities (e.g., watching television, eating junk food) than when they were depicted engaging in stereotype inconsistent behaviors (e.g., exercising or preparing vegetables), suggesting a stronger mental link between being overweight and being lazy or unhealthy [22]. In another study, body-related worrying was associated with overweight individuals’ negative affect, whereas overweight individuals without body-related worrying had significantly more positive affect [23]. This suggests that the amount of concern about one’s weight rather than body weight itself might moderate overweight individual’s mood. Also, fat stigma and “fat jokes” are considered more socially acceptable than other prejudicial jokes (e.g., race, sex, religion), making it more commonplace and normalized to make weight jokes and, for overweight individuals, to embrace such jokes and endorse them [24].

Thus, a critical discrepancy exists between how overweight individuals are perceived to feel and their actual reactions to experiences. Assessing the emotionality and mood of others is a necessary, frequent occurrence in everyday human life. Many socially-relevant judgments are made from processing an individual’s emotional expressions, such as approachability, trustworthiness, and friendliness [25, 26]. Social judgments from facial expressions do not require long and elaborate processing and interpretation times, and can in fact be made very quickly from only brief exposures [27]. Because perceptive interpretations strongly inform and motivate behavior, it is important to understand the factors that may influence the assessment of an emotion. Recent research found that the age, sex, or race of a face systematically biased how emotional expressions are perceived in a manner that matched with stereotype-driven impressions [28–30]. Specifically, older faces were more frequently perceived as having a happy expression and less frequently perceived as having an angry expression than younger faces [28]. Compared to neutral male faces, neutral female faces were rated as more fearful, happy, and less angry [29]. Similarly, participants with implicitly reported racial prejudices were more likely to label a black face as being angry and for a wider range of emotional variability than compared to white faces [30]. Also, the context surrounding a facial image, such as background pictures, stories, or scenarios, has been shown to influence judgments on the emotion a face is thought to portray [31–33]. In all these cases, psychosocial and contextual factors which have no direct relevance to emotion have been shown to systematically bias the judgment of facial expressions. Thus, despite the strong social cues that can be given by a facial expression, there exists a subjective biasing component in interpreting the emotion of a face. In the present study, we sought to explore whether the weight (fatness) of a face might also skew the perception of an emotional expression. Given that the stereotyped perception of overweight individuals as happy is, in fact, contrary to clinical evidence about low self-esteem and depression in overweight adults, it is critical to explore this phenomenon further to see how weight stereotypes might influence emotion judgments.

The stereotypes that surround being fat or overweight may impact how others perceive someone’s emotional state more than we realize. Because emotional perception provides critical information for driving behavioral responses, it is important to examine how emotion-irrelevant factors, such as facial weight, might systematically bias perceptions of emotion. The present study implemented a two-alternative, forced-choice task across two experiments to explore the influence of weight variations on the *subjective perception decision threshold* of emotional judgments on a continuum of emotional expressions (i.e., neutral face to 100% happy face; neutral face to 100% sad face). This forced-choice paradigm has been successfully used in previous facial emotion studies [28, 34–36]. In the first experiment, participants were asked to categorize the emotion of healthy or overweight faces with varying levels of happiness (0% to 100%) into either neutral or happy categories. Happiness was chosen as the experimental emotional expression condition specifically because of its stereotypical association with obesity [16, 17]. In the second experiment, participants were asked to categorize the emotion of healthy or overweight faces with varying levels of sadness (0% to 100%) into either neutral or sad categories. We chose sadness in this experiment in order to look at the effect that the emotion considered to be opposite of happiness might have. We expected that the perceptual threshold for selecting sad and happy as the emotion expressed would be systematically biased by the weight of the facial stimuli shown. More specifically, we hypothesized that the task-irrelevant factor of a face’s weight would increase the perceptual decision threshold for “sad” selections (i.e., less “sad” selections for overweight faces) and decrease the threshold for “happy” selections (i.e., more “happy” selections for overweight faces) in a systematic way due to weight biases impacting participants’ affective perceptions. That is to say, we predicted that overweight faces would be more likely to be judged as happy and less likely judged as sad than healthy weight faces. We also aimed to explore whether explicit attitudes about being overweight or one’s own weight would impact the perceptual decision threshold for emotional categorization by examining the relationship between scores on the Anti-Fat Attitudes (AFA) questionnaire [37] and BMI with subjective perceptual threshold shift values. In particular, considering past findings of an association between body-related worrying and negative affect for overweight individuals [23], we intuitively expected that a “negative attitude toward becoming fat” or one’s own body mass might *counteract* the stereotypical decision threshold shifts (i.e., “fat as more happy and less sad”) of affective perception in our experiments.

## Materials and Methods

### Participants

Sixty-four healthy college students (*M =* 23.6 years old, *SD =* 7.5 years; 23 males; 43 Caucasian, 3 Hispanic, 8 African American, 8 Asian, and 2 other) were recruited though the Psych Pool online research recruitment system at the University of Missouri—Kansas City (UMKC). Participation took place individually, in person, and compensation for completion was course credits. All experimental protocols were reviewed and approved by the Institutional Review Board of UMKC (IRB SS 13–795). Upon giving written informed consent, participants had their height and weight measured to calculate Body Mass Index (BMI; kg/m^2^; Mean BMI = 24.1, *SD* = 4.7). Participants were randomly assigned to one of two experimental paradigms. Thirty-two participants completed experiment 1 in which they were asked to rate experimental faces as either happy or neutral, and another 32 participants completed experiment 2 in which they were asked to rate experimental faces as sad or neutral. There was no significant difference in age, sex, ethnicity, and BMI between the two groups of participants (all *p* > .05).

### Self-Report Measure

Participants self-reported their anti-fat attitudes using the Anti-fat Attitudes (AFA) questionnaire [37]. The 13-item AFA measure includes three subscales, namely, “Fear,” “Willpower” and “Dislike.” The “Fear” subscale assesses an individual’s fear that they themselves may become fat (e.g., I worry about becoming fat). The “Dislike” subscale measures participant’s subjective distaste for fat individuals (e.g., I really don’t like fat people much). The last subscale, “Willpower,” measures beliefs that being fat is a result of a lack of self-control (e.g., some people are fat because they have no willpower). Responses are assessed on a nine-point Likert-type scale, with higher scores representing larger anti-fat prejudice. In our data, the observed Cronbach’s alpha values of the “Fear,” “Dislike,” and “Willpower” subscales were .83, .82, and .61, respectively.

### Stimuli

**Fig 1:**
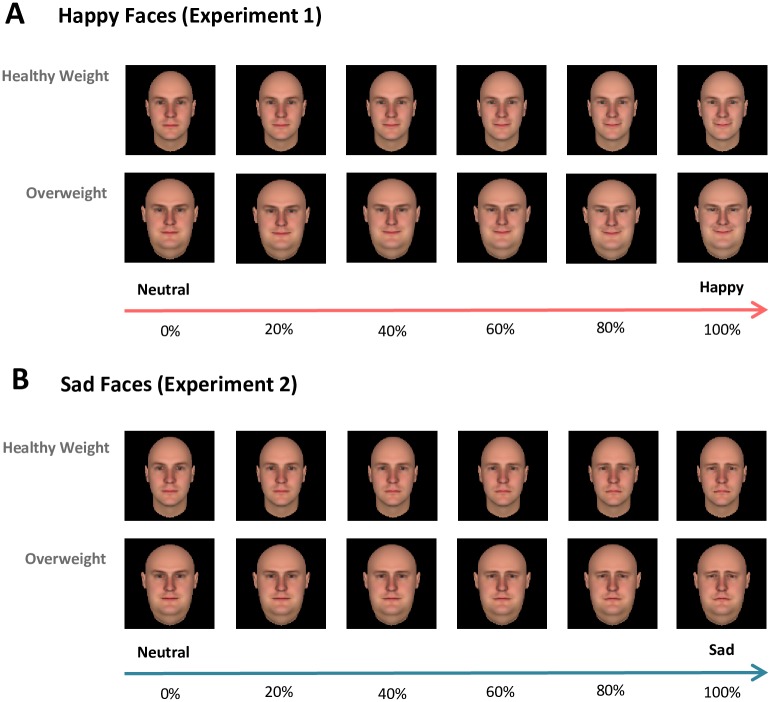
Experimental stimuli. A. Exemplar facial stimuli used for the neutral-happy judgment task. B. Exemplar facial stimuli used for the neutral-sad judgment task. All facial stimuli were computer-generated and no actual faces were used in our study. A total of four computer-generated identities (two males and two females) were used in each experiment. Emotional expression and bodyweight of facial stimuli were systematically manipulated by using a morphing software. Faces have emotion gradients ranging from 0% (neutral emotion) to 100% (full emotion; either happy or sad) by increments of 20%.

All facial stimulus identities were constructed using FaceGen Modeller Software (Singular Inversions, Toronto, ON, Canada) from the computer-generated face set available with the software. The FaceGen’s face model is based on a 3D laser-scanned face database, and it allows for parametrically adjusting faces on multiple dimensions including emotional expression and body weight. The weight category of a face was distinguished by manipulating the facial structure of the original (i.e., healthy weight) face to have wider cheek, facial features, and skull structures for the overweight version by using the morphing function integrated in the FaceGen Modeller software. The same manipulation procedure was used for each of the four identities for consistency. Then, Fantamorph software (Abrosoft, Lincoln, NE, USA) was utilized in order to systematically create a gradient for the emotional intensities of the faces. The computer-generated facial stimuli consisted of four facial identities (two males and two females) with two different weight levels (overweight and healthy weight) across an emotional gradient of six different emotional levels. Emotions ranged from 0% (neutral) to 100% (extreme affect) separated by 20% intervals of either increasingly happy expressions (Experiment 1) or increasingly sad expressions (Experiment 2). This resulted in 48 unique faces (4 identities x 2 weight levels x 6 emotional levels) for each task (see Fig 1 for example). Separate stimulus sets (one male and one female) were created for a short practice session.

### Experimental Tasks

A novel, two-alternative forced choice (2-AFC) emotional judgment task similar to the authors’ judgment task described in previous literature [28] was created for this study. We designed and deployed our task using SuperLab software (Cedrus Corporation, San Pedro, CA). Participants assigned to Experiment 1 (neutral-happy judgment task) completed a task designed to measure how the body weight of a facial stimulus would change their perceptual decision of whether the emotional expression was “neutral” or “happy,” while participants assigned to Experiment 2 (neutral-sad judgment task) completed a task measuring how the body weight of a facial stimulus impacted their perceptual decision of whether the emotional expression was “neutral” or “sad.” Structurally and procedurally, the two experiments were identical except for the type of emotional judgment (neutral or happy; neutral or sad). We separated our experiments into two due to the relatively long duration of our emotional judgment task (~ 60 min).

**Fig 2:**
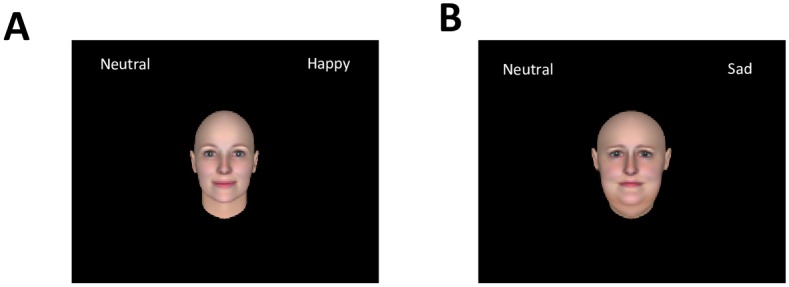
Affective perceptual judgment task. A. Sample screen of the neutral-happy judgment task (Experiment 1). B. Sample screen of the neutral-sad judgment task (Experiment 2). Participants were asked to make perceptual judgments about the emotion of faces (neutral vs. happy; neutral vs. sad) in a two-alternative forced-choice procedure. The spatial locations of emotion category labels were counterbalanced across participants.

In each experiment, participants made emotion judgments on a randomized order of healthy weight and overweight faces at each level of emotional expressions. The two decision categories for participants were determined by the experimental task to which they had been randomly assigned (see Fig 2A for Experiment 1 and Fig 2B for Experiment 2). Before the main task began, participants engaged in a short practice task (24 trials) in order to become familiar with the parameters of the experiment. At the beginning of each trial, participants were shown a black background screen with a white central fixation-cross and two emotion category labels (one in the upper-left and one in the upper-right corner). The spatial position (left or right) of category labels was counterbalanced across participants. After a random interval delay (1~2 s, with 50 ms variations), a facial image (400 by 400 pixels) was displayed in the center of the screen for 100 ms. The brief facial stimulus presentation was chosen to eliminate or minimize the occurrence of deliberate eye saccades [38], as similarly done in previous perceptual decision studies [28, 34–36, 39]. Our intent was to demand the participant focus on the emotion judgment itself to minimize confounds in judgments and to control for deliberate redirection of attention elsewhere on the screen that may lead to distraction. As previous literature has shown, social judgments made from brief exposures to a face are considered as reliable as judgments made after longer exposures [27]. Participants were asked to sort the facial expression of each stimuli into one of two categories as quickly and accurately as possible by pressing one of assigned keyboard buttons (“e” for the upper-left hand category, and “i” for the upper-right hand category). After a response was inputted, a yellow fixation-cross appeared in the center of the screen for 500 ms in order to indicate that the response was registered. If the participant failed to sort a face within the 2 sec time constraint, the word “MISS” appeared in red on the screen for 500 ms. Participants completed a total of 960 trials (2 body weight groups × 2 sexes × 6 emotion intensity levels × 40 repetitions) across 4 separate blocks. Between each block, text appeared on the screen informing the participant that the previous block had been completed and that they were allowed a short break before moving on to the next block. The experimental task took approximately one hour. Split-half reliability measures showed high-level consistency for both emotion judgment tasks (*r* = .96 for happy decisions; *r* = .94 for sad decisions).

### Psychometric Curve Fitting

We hypothesized that the body weight of facial stimuli would bias the emotion judgment (neutral or happy; neutral or sad) on parametrically morphed emotional faces by systematically shifting the shape of the psychometric decision functions. Following a nonlinear psychometric curve-fitting approach that has been successfully employed in previous emotion research [28, 34–36], including our own where we similarly described this process in detail [28], we fitted psychometric curves to our behavioral data (i.e., the proportion of “Happy” or “Sad” decisions in each different level of emotional intensities of the healthy weight and overweight faces) by using the Naka-Rushton contrast response model [40, 41] with an *OLS* (Ordinary Least Square) criterion.

$$response=\frac{R_{\text{max}}\times C^{n}}{C^{n}+C_{50}^{n}}+M$$

Here, *response* represents the proportion of “Happy” (Experiment 1) or “Sad” (Experiment 2) decisions, *C* is the graded emotional intensity levels of the healthy weight and overweight faces (contrast: 0% ~ 100% happy expression or 0% ~ 100% sad expression, in 20% increments, *n* is the exponent that determines the slope of the function, and *R*_*max*_ is the asymptote of the response function, while *M* is the response at the lowest stimulus intensity. Most importantly, *C*_*50*_ indicates the stimulus intensity at which the response is half-maximal (also called the “threshold” or “Point of Subjective Equality: *PSE*”), which were used for our hypothesis tests. Because the proportions (0 ~ 1 range) were entered as input data, we constrained the *R*_*max*_ parameter to be equal to or less than 1, and the *M* parameter to be equal to or larger than 0 in our data fits. We fitted psychometric curves separately for each body weight condition (Experiment 1, healthy weight and overweight happy faces; Experiment 2, healthy weight and overweight sad faces). Curve fitting procedure was performed by using GraphPad Prism software (GraphPad Software, La Jolla, CA).

**Fig 3:**
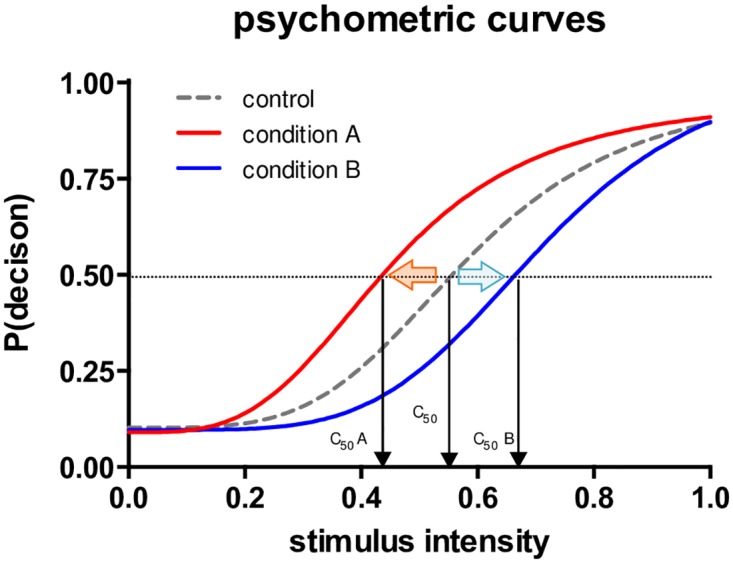
Psychometric response curve modeling by Naka-Rushton contrast response function. X-axis represents stimulus intensity level and Y-axis represents response probability. The stimulus intensity in this study represents the incremental increase of emotional intensity of facial expressions. The response represents the proportion of happy (experiment 1) or sad (experiment 2) decisions in a two-alternative forced choice task. The *C*_*50*_ or *PSE* (Point of Subjective Equality) parameter indicates the perceptual decision threshold. A leftward shift of the psychometric curve (red arrow) would constitute evidence for a decreased perceptual threshold for condition A compared to the control condition, and a rightward shift of the psychometric curve (blue arrow) would constitute evidence for an increased perceptual threshold for condition B compared to control condition, respectively.

We predicted that body weight would influence affective perception of the emotional expressions of happiness and sadness in faces by systematically changing the decision threshold (*PSE*) that is represented by the *C*_*50*_ parameter. As illustrated in Fig 3, the changes of threshold are often described by a leftward shift (decrease of decision threshold) or a rightward shift (increase of decision threshold) of psychometric curves by the contrast gain model in visual perception research [42–44] and has been reported in previous studies of affective perception of facial stimuli, including our own, similar study on facial age [28, 34–36]. In our experimental context, we predicted a decreased happy decision threshold (*C*_*50 happy*_) for overweight faces compared to healthy weight faces (a leftward horizontal shift; more sensitive happy perception for overweight faces) and an increased sad decision threshold (*C*_*50 sad*_) for overweight faces compared to healthy weight faces (a rightward horizontal shift; less sensitive sad perception for overweight faces), matching to stereotype-driven social impressions [28–30].

## Results

### Emotion Judgment

To test our research hypotheses, the effect of task-irrelevant body weight of faces (healthy weight and overweight) on the affective judgment (Experiment 1: neutral vs. happy; Experiment 2: neutral vs. sad) of facial stimuli was systematically examined by employing both repeated-measures ANOVAs and nonlinear psychometric curve fitting approaches. Trials in which participants failed to give a response in the time allotted (2 s) were treated as missing data and excluded from analysis. Among 960 trials, participants missed an average of 11.2 trials (1.2%) in Experiment 1, and an average of 8.0 trials (0.8%) in Experiment 2. There was no systematic effect of Experiment Type, *F*(1,62) = 1.31, *p =* .26, Bodyweight Groups, *F*(1,62) = 0.42, *p =* .52, or Emotion Intensity, *F*(5,310) = 0.25, *p =* .94.

**Fig 4:**
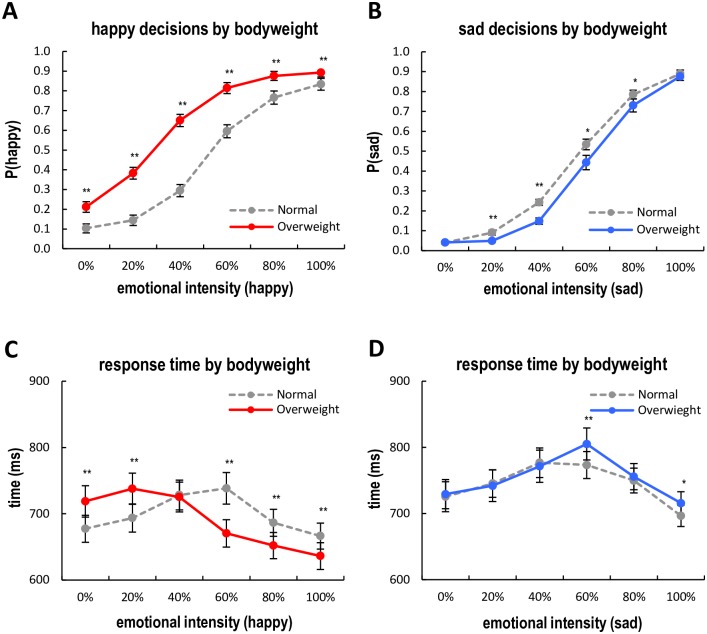
Behavioral findings. A. Average probability of happy decisions as a function of bodyweight and emotional intensity of faces (Experiment 1). B. Average probability of sad decisions as a function of bodyweight and emotional intensity of faces (Experiment 2). C. Response times of happy decisions (Experiment 1). D. Response times of sad decisions (Experiment 2). Error bars denote the standard error of the mean. * *p* < .05, ** *p* < .01.

For happy (Experiment 1) and sad (Experiment 2) expression judgment tasks, we separately performed 2 (Bodyweight: Healthy weight faces, Overweight faces) by 6 (Emotion Intensity: 0% ~ 100% in 20% increments) repeated-measures ANOVAs on the behavioral data of proportions of happy or sad decisions. Means and standard deviations are shown in Table 1. The ANOVA result on happy decisions showed a significant 2-way interaction effect of Bodyweight x Emotion Intensity, *F*(5,155) = 32.55, *p* < .001, partial *η*^*2*^ = .51, as well as main effects of Bodyweight, *F*(1,31) = 294.76, *p* < .001, partial *η*^*2*^ = .91, and Emotion Intensity, *F*(5,155) = 214.38, *p* < .001, partial *η*^*2*^ = .87. For simple effect analyses, we performed a series of paired *t*-tests for each level of Emotion Intensity. As shown in Fig 4A, across all levels of happy expressions (0% to 100%), overweight faces were more frequently perceived as having a happy expression than healthy weight faces, *t*(31) = 7.26, *p* < .001, *d* = 1.29; *t*(31) = 11.68, *p* < .001, *d* = 2.06; *t*(31) = 12.00, *p* < .001, *d* = 2.12; *t*(31) = 9.65, *p* < .001, *d* = 1.71; *t*(31) = 6.55, *p* < .001, *d* = 1.15; *t*(31) = 3.98, *p* < .001, *d* = 1.15, suggesting a systematic perceptual bias toward positive emotional expression perception of overweight faces that have a varying degree of neutral to happy expressions. Similarly, the ANOVA result on sad decisions showed a significant 2-way interaction effect of Bodyweight x Emotion Intensity, *F*(5,155) = 6.14, *p* < .001, partial *η*^*2*^ = .17, as well as main effects of Bodyweight, *F*(1,31) = 13.65, *p* < .001, partial *η*^*2*^ = .31, and Emotion Intensity, *F*(5,155) = 745.54, *p* < .001, partial *η*^*2*^ = .96. Interestingly, subsequent simple effect analyses on sad expressions revealed findings that contrast with those of the happy expressions. As shown in Fig 4B, overweight faces were less frequently perceived as sad than healthy weight faces at the 20%, 40%, 60%, and 80% sad expressions, *t*(31) = -3.60, *p* < .01, *d* = 0.64; *t*(31) = -5.95, *p* < .001, *d* = 1.05; *t*(31) = -2.74, *p* < .05, *d* = 0.48; *t*(31) = -2.10, *p* < .05, *d* = 0.37, whereas there was no significant difference at the extreme levels (0% and 100% sad faces), all *p* > .05. Again, these results indicate that participants showed a systematic perceptual bias toward positive (i.e., less negative) emotional expression perception of overweight faces that have a varying degree of neutral to sad expressions. Given that our sample comprised an uneven number of males (*n* = 41) and females (*n* = 23), we performed an additional repeated-measures ANOVA by adding Gender as an additional variable to rule out a potential confounding effect. As expected, our post hoc analysis did not show any significant main or interaction effect with other factors (Experiment Type, Bodyweight, and Emotion Intensity), all *p* > .05.

**Table 1:** Means and standard deviations of the proportion of happy and sad decisions.

	Emotion Intensity Level of Morphed Faces					
Face Type	0%	20%	40%	60%	80%	100%
Happy Decisions (Experiment 1)						
Healthy Weight	.103 (.127)	.144 (.150)	.295 (.172)	.595 (.185)	.766 (.191)	.833 (.168)
Overweight	.211 (.152)	.383 (.171)	.650 (.177)	.814 (.157)	.876 (.127)	.892 (.127)
Sad Decisions (Experiment 2)						
Healthy Weight	.041 (.040)	.091 (.069)	.243 (.087)	.534 (.149)	.785 (.127)	.889 (.110)
Overweight	.041 (.041)	.049 (.044)	.149 (.091)	.443 (.204)	.730 (.188)	.876 (.114)

Next, we conducted similar 2 (Bodyweight) by 6 (Emotion Intensity) repeated-measures ANOVAs on the response time data for happy or sad decisions. Means and standard deviations of the response times are shown in Table 2. For the happy-expression judgment task, the ANOVA result showed a significant interaction effect of Bodyweight x Emotion Intensity, *F*(5,155) = 22.79, *p* < .001, partial *η*^*2*^ = .4241. Main effects of Bodyweight, *F*(1,31) = 6.83, *p* < .05, partial *η*^*2*^ = .18, and Emotion Intensity, *F*(5,155) = 18.19, *p* < .001, partial *η*^*2*^ = .37, were also significant. As shown in Fig 4C, subsequent simple effect analyses showed that the perceptual decision time for overweight faces was slower than for healthy weight faces at the lower levels of happy expressions (0% and 20%), *t*(31) = 5.21, *p* < .001, *d* = .79; *t*(31) = 4.45, *p* < .001, *d* = .73, while decision time for overweight faces was faster than healthy weight faces at the higher levels of happy expressions (60%, 80%, and 100%), *t*(31) = -5.42, *p* < .001, *d* = .96; *t*(31) = -4.23, *p* < .001, *d* = .75; *t*(31) = -4.86, *p* < .001, *d* = .86. Similarly, the ANOVA result on the response times in the sad expression judgment task revealed a significant interaction effect, *F*(5,155) = 3.77, *p* < .001, partial *η*^*2*^ = .11, a main effect of Bodyweight, *F*(1,31) = 4.74, *p* < .05, partial *η*^*2*^ = .13, and a main effect of Emotion Intensity, *F*(5,155) = 18.20, *p* < .001, partial *η*^*2*^ = .37. The simple effect analyses showed that the perceptual decision time for overweight faces was slower than for healthy weight faces at the 60% and 100% level sad expressions, *t*(31) = 3.53, *p* < .01, *d* = .62; *t*(31) = 2.68, *p* < .05, *d* = .47, whereas there was no significant difference at the other levels of sad expressions, all *p* > .05 (see Fig 4D).

**Table 2:** Means and standard deviations of the response time in milliseconds.

	Emotion Intensity Level of Morphed Faces					
Face Type	0%	20%	40%	60%	80%	100%
Happy Decisions (Experiment 1)						
Healthy Weight	677 (115)	694 (120)	728 (127)	738 (135)	686 (116)	666 (110)
Overweight	719 (134)	738 (133)	725 (127)	670 (117)	652 (112)	636 (114)
Sad Decisions (Experiment 2)						
Healthy Weight	726 (128)	746 (118)	777 (126)	773 (115)	750 (106)	670 (91)
Overweight	729 (125)	742 (135)	771 (137)	805 (137)	756 (112)	716 (97)

### Perceptual Decision Threshold

As discussed in the introduction, we speculated that the body weight of target face stimuli would systematically influence the perceptual decision threshold of emotional expression judgment. More specifically, we hypothesized that participants would show a *decreased* perceptual decision threshold for positive emotion (i.e., more sensitive/frequent happy decisions) for overweight faces, but they would show an *increased* perceptual threshold for negative emotion (i.e., less sensitive/frequent sad decisions). This corresponds to a systematic perceptual bias toward positive emotional expression perception of overweight faces in both positive and negative emotion domains of varying degrees of emotional valence intensity (neutral ~ happy; neutral ~ sad). In our 2-AFC perceptual decision tasks, the perceptual decision threshold or *PSE* (Point of Subjective Equality) that determines binary responses (i.e., neutral vs. happy; neutral vs. sad) was indexed by estimating *C*_*50*_ parameters (i.e., the emotional intensity values in the x-axis that produce 50% happy or sad decisions; see Fig 3) from choice data. We tested our experimental hypothesis by fitting the Naka-Rushton contrast response function for each individual’s data and then comparing the estimated *C*_*50*_ parameters between healthy weight and overweight faces in group-level analyses. The estimated best-fit values and standard errors of the Naka-Rushton contrast response model parameters are shown in Table 3.

**Table 3:** Means and standard deviations of best-fit values of psychometric curve fit parameters.

Face Type	*C*_*50*_	*R*_*max*_	*n*	*M*
Happy Decisions (Experiment 1)				
Healthy Weight	.570 (.151)	.813 (.244)	4.877 (2.041)	.108 (.120)
Overweight	.402 (.221)	.759 (.221)	3.183 (1.593)	.218 (.148)
Sad Decisions (Experiment 2)				
Healthy Weight	.602 (.081)	.950 (.105)	4.174 (1.120)	.062 (.045)
Overweight	.658 (.124)	.939 (.084)	5.611 (1.204)	.049 (.041)

**Fig 5:**
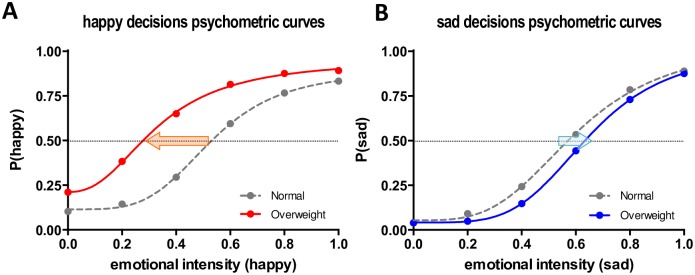
Psychometric curve fits. A. Happy decisions psychometric curves by body weight (Experiment 1). B. Sad decisions psychometric curves by body weight (Experiment 2). For each bodyweight condition, psychometric curves were separately fitted by using the Naka-Rushton response function. Compared to healthy weight faces (gray dashed line), a leftward-shift of the psychometric curve of overweight faces (red line) in the neutral-happy judgment task and a rightward-shift of the psychometric curve of sad faces (blue line) in the neutral-sad judgment task were observed. A dotted horizontal line represents the 50% probability of a happy or sad decision.

For the happy-expression judgment task (Experiment 1), the means of the *C*_*50*_ parameter for healthy weight faces and overweight faces were .570 (*SD* = .151) and .402 (*SD* = .221), respectively. As expected, the perceptual decision threshold for happy-expression judgment was significantly lower for overweight faces compared to healthy weight faces, *t*(31) = -4.70, *p* < .001, *d* = .83 (see a leftward shift of Fig 5A). In other words, as the *C*_*50*_ parameters indicate, in the happy-expression judgment task participants required only 40.2% emotion intensity to make a happy decision for overweight faces, while they required 57.0% emotion intensity level for healthy weight faces. The average decrease of perceptual decision threshold for happy expression judgment was -16.8% (95% *CI*: -9.5 ~ -24.0%). On the other hand, the means of the *C*_*50*_ parameter for healthy weight faces and overweight faces were .602 (*SD* = .081) and .658 (*SD* = .1240) for the sad-expression judgment task (Experiment 2). Again, as we hypothesized, the perceptual decision threshold for sad-expression judgment was significantly higher for overweight faces compared to healthy weight faces, *t*(31) = 2.39, *p* < .05, *d* = .42 (see a rightward shift of Fig 5B). In the sad expression judgment task, participants required 65.8% emotion intensity level to make a sad decision for overweight faces, while they required 60.2% emotion intensity level for healthy weight faces. The average increase of perceptual decision threshold for sad-expression judgment was +5.5% (95% *CI*: +0.8 ~ +10.3%). To check the robustness of findings, we also compared two *C*_*50*_ parameters of healthy weight faces between two separate experiments, which did not show a significant difference, *t*(62) = 1.07, *p* > .05. Overall, these results imply that participants more sensitively perceived positive emotional expressions of overweight faces compared to healthy weight faces, while they less sensitively perceived negative emotional expressions of overweight faces compared to healthy weight faces.

### Correlation Analysis

**Fig 6:**
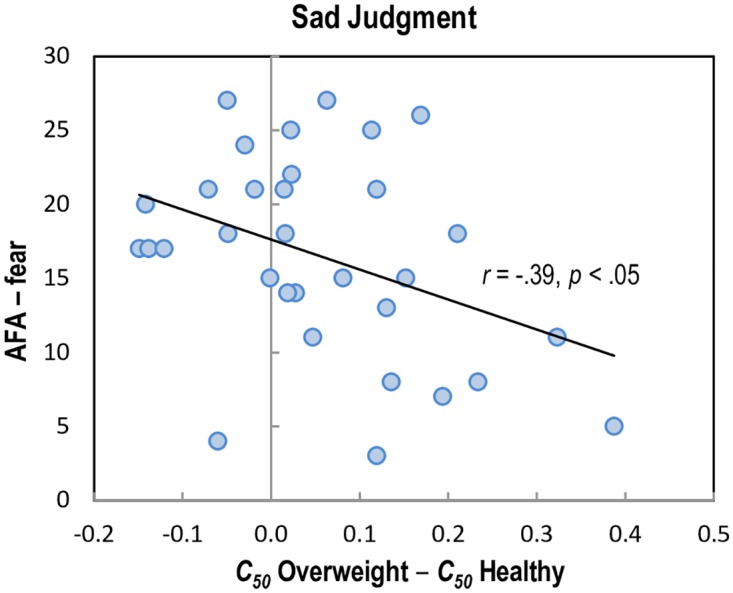
Correlation result. A scatter plot of the relationship between AFA (Anti-Fat Attitude)–fear scale and *C*_*50*_ parameter differences (*C*_*50*_ Overweight − *C*_*50*_ Healthy weight) in the neutral-sad judgment task. Higher *C*_*50*_ difference represents larger bodyweight modulation (the rightward shift of the psychometric curve) on sad decisions. The solid line represents a linear fit.

We hypothesized that the perceptual decision threshold changes by emotional expressions could be related to individuals’ BMI score and/or their attitudes toward overweight individuals. To investigate this possibility, we conducted exploratory correlational analyses using *C*_*50*_ difference scores (indexed by *C*_*50*_ overweight − *C*_*50*_ healthy weight differences for each experiment). The BMI scores did not show a significant effect in both conditions, all *p* > .05, indicating that individuals’ own body masses were not associated with the perceptual decision threshold shift for overweight emotional faces. However, as shown in Fig 6, the decision threshold shift of sad judgments by body weight was negatively correlated with the AFA fear scale, *r*(30) = -.39, *p* < .05, suggesting that individuals who have a psychological fear or worry about becoming fat show smaller stereotypical changes in their decisions, while individuals who do not have this fear show larger stereotypical changes (i.e., judging fat as less sad). For the other scales of the AFA measures, we could not observe any significant correlation.

## Discussion

Body weight serves as an important social cue that can impact behaviors and treatment towards an individual. Although research has shown that overweight individuals tend to have higher levels of depression, lower self-esteem, and higher perceived stress [12–14], overweight individuals are often stereotyped by others as being happy-go-lucky and carefree [16, 17, 24]. Due to the stereotypical association between fatness and jolliness, overweight individuals’ emotions may not be properly interpreted in their social relationships, potentially causing additional disadvantages, psychological burdens, or, conversely, social benefits. Given the relevance of weight globally, but particularly in American society, where approximately two-thirds of individuals fall into the categories of “overweight or obese” [1], we were interested in exploring whether people show a bias in interpreting the emotional expressions of overweight individuals compared to healthy weight individuals. The primary purpose of this study was to investigate how the weight of the faces, which is an irrelevant factor in determining emotional state, influences the perceptual judgment of positive and negative facial expressions. Using gradually morphed neutral to happy and neutral to sad facial expressions across both healthy weight and overweight faces, we examined how the weight of the faces systematically influenced the affective perception of facial expressions. The 2-AFC experimental paradigms allowed us to determine the effect of weight (overweight vs. healthy weight) in facial stimuli on the subjective perceptual decision threshold of affective perception.

We hypothesized that the task-irrelevant factor of weight would influence participants’ emotional judgments for facial expressions in a way consistent with the “jolly-fat” stereotyped perception of overweight individuals as happy and not sad. Specifically, we predicted that overweight faces would be more often judged as happy at the same levels of emotional expressivity than healthy weight faces and less often judged as sad than healthy weight faces in the same levels of emotional expressivity. That is to say, we anticipated that the addition of appearing overweight would *decrease* the observer’s subjective perceptual decision threshold for happy expressions (i.e., increasing the number of “happy” decisions in Experiment 1) and *increase* the observer’s subjective perceptual decision threshold for sad expressions (i.e., reducing the number of “sad” decisions in Experiment 2). Our research hypotheses were strongly supported in both experiments. In Experiment 1, overweight faces had a significantly increased number of “happy” decisions when presented at every happy level (0%, 20%, 40%, 60%, 80% and 100%) compared to healthy weight faces. In Experiment 2, overweight faces had a significantly reduced number of “sad” decisions when presented at the ambiguously sad levels (20%, 40%, 60%, and 80%) compared to healthy weight faces. More specifically, the subjective perceptual decision threshold for categorizing a face as expressing happy emotion was significantly lower for overweight faces than for healthy weight faces (i.e., a leftward horizontal shift of the psychometric curve; Fig 5A), whereas the subjective perceptual decision threshold for categorizing a face as expressing sad emotion was significantly higher for overweight faces than for healthy weight faces (i.e., a rightward horizontal shift of the psychometric curve; Fig 5B). Although it could be argued that the bias towards labeling an overweight face as happy was due to the wider structure of the overweight faces, which mimics the structure of a smiling face, our opposite results in the sad/neutral judgment task would seem to counteract this point. That is to say, we believe the weight of the face itself was the factor influencing the emotional judgment bias towards calling an overweight face, happy, because the direct opposite findings held true for sad emotional expressions, which tend not to impact the overall shape of a face when expressed. The reason that weight, an otherwise irrelevant factor to the judgment of emotional expression, had a systematic impact on happy and sad categorization was not directly assessed in this study; however, based on previous literature linking a stereotypic association between being overweight and being happy, we believe that these implicit social opinions played a role in the pattern of responses we observed. However, although we did our best to experimentally control other facial features by using computer-generated faces, it might be still possible that some perceptual features played a role to a certain extent in our experiments.

Additionally, we speculated that the time it takes to judge the emotion of a face would be longer for faces that require a decision inconsistent with our hypotheses (e.g., a sad, overweight face or a happy, healthy weight face) due to additional cognitive load demands of processing counter-stereotype information. Our results indicated that this was indeed the case, with an interaction of body weight and emotional intensity on reaction times. Specifically, perceptual decision time for overweight faces was slower than healthy weight faces at the lower levels of happy expressions (0% and 20%), but faster than healthy weight faces at the higher levels of happy expressions (60%, 80%, and 100%). This was partially supported with the sad experiment results as well, which found that the decision time for overweight faces was slower than healthy weight faces at the 60% and 100% level sad expressions. The findings from both experiments would suggest that participants used more cognitive resources—and thus took more time—when deciding how to categorize stereotype-inconsistent judgments as compared to consistent judgments. Judging an overweight face as happy or, conversely, a healthy weight face as sad required less decision time than the opposite pairing because it was in agreement with cognitive stereotypes.

We also explored whether a participant’s own body weight or their attitudes about being overweight correlated with their performance on the emotional judgment task. Our results showed that participants’ psychological attitude toward obesity rather than their own body mass was an important factor in our experimental outcomes. The BMI scores were not correlated with the decision threshold shifts in both experiments, indicating that one’s own physical body mass is not a critical variable to determine the subjective decision threshold change. This lack of relationship may be due, in part, to limitations in measuring body weight healthiness and overweightness restricted by the calculation of BMI as a ratio of weight and height, without considering bone density, muscle mass, and other components that would influence overall healthiness. On the contrary, the decision threshold shift of sad judgments by body weight (Experiment 2) revealed a significant negative correlation with the AFA fear scale, suggesting that individuals who do not worry about becoming fat show larger stereotypical decision changes. Conversely, we speculate that those with a greater fear of becoming fat themselves may have been more sensitive to the weight-levels of others, and thus showed less of a bias when judging overweight faces’ sadness because of their negative feelings towards personal fatness. This mechanism might be relevant to the self-serving or self-weight biases often discussed in obesity literature [45–47]. However, we did not observe a significant correlation in happy judgment (Experiment 1). Thus, as this is the first study of its type, it is somewhat difficult to confidently draw conclusions on our exploratory correlation analysis results. Further replications will be required to confirm our findings.

Our study contains several limitations worth noting. First, we used computer-generated facial stimuli in place of real-world facial stimuli in our judgment task, which has been successfully employed in previous research [48, 49]. This allowed us to systematically manipulate the weight of identical faces as well as to increase the amount of emotionality expressed in the face in equal intervals and while controlling for other facial variants (e.g., individual differences in facial features), which is not feasible with natural, real-word facial stimuli. Indeed, to our knowledge there exists no face database of the same face identity photographed across multiple body weights and with varying facial expressions that would have suited our research purpose. Although our experimental manipulation could increase the interval validity, it is worth noting that it may have reduced the external realism or generalizability. Second, it is also important to consider that our study only explored the effect of body weight on affective perception for happiness and sadness. Thus, the interpretation of the results should be limited to only these two emotions. However, it might be possible that other positive or negative expressions such as contempt, surprise, anger, disgust, or fear have similar effects in a more general way, if they share similar stereotypical associations with being overweight in our society. Further research should be done to determine the impact of body weight on different types of emotions. Another limiting factor was the sample size for the experiments (32 participants for each experiment), which makes it challenging to confidently draw conclusions on the correlation analysis results by our study alone.

Conveying accurately how an individual feels to others is critical for interpersonal interactions. But, overweight individuals often receive stereotyped weight-related commentary in their social lives, even when they do not intend it. Our research experimentally demonstrated that an incidental factor such as body weight could have a systematic effect on emotional perception and judgment, which may serve to fulfill stereotyped social perception. The impact of weight stigma, which has already been shown to be widespread [3], may also show through in the perception of emotional expressions, which in turn impact with whom we may choose to interact. To some extent, being seen as more likely happy and less likely sad due to a bias in the assessment of emotional expression in overweight individuals may be socially beneficial and encourage more pro-social behavior in others, but it may also be seen as disadvantageous if it interferes with conveying one’s true emotions or mood. For example, inaccurate assessment of an overweight individuals’ emotional expression as more positive and less negative may impede social gestures common when sadness is perceived in others. In other words, this may mean it is less likely that someone would reach out to a sad, overweight individual in response to assessing his or her sad expression because of a perceptual bias towards a more neutral interpretation. Because weight is both a highly salient and socially relevant feature, it would be important to explore from where this bias originated and how this bias would be effectively intervened upon in future studies.
